# Insufficient Stability of Clavulanic Acid in Widely Used Child-Appropriate Formulations

**DOI:** 10.3390/antibiotics10020225

**Published:** 2021-02-23

**Authors:** Ines Mack, Mike Sharland, Janneke M. Brussee, Sophia Rehm, Katharina Rentsch, Julia Bielicki

**Affiliations:** 1 Paediatric Infectious Diseases, University Children’s Hospital Basel, 4056 Basel, Switzerland; julia.bielicki@ukbb.ch; 2 Paediatric Infectious Diseases Research Group, Institute for Infection and Immunity, St George’s University of London, London SW17 0QT, UK; msharland@sgul.ac.uk; 3 Paediatric Pharmacology and Pharmacometrics, University Children’s Hospital Basel, 4056 Basel, Switzerland; jantinebrussee@gmail.com; 4 Medical Parasitology and Infection Biology, Swiss Tropical and Public Health Institute, University of Basel, 4051 Basel, Switzerland; 5 Laboratory Medicine, University Hospital Basel, 4031 Basel, Switzerland; sophiasibylla.rehm@usb.ch (S.R.); Katharina.Rentsch@usb.ch (K.R.)

**Keywords:** amoxicillin-clavulanic acid, clavulanate, degradation, child-appropriate formulation

## Abstract

Amoxicillin-clavulanic acid (AMC) belongs to the WHO Essential Medicines List for children, but for optimal antimicrobial effectiveness, reconstituted dry powder suspensions need to be stored in a refrigerated environment. Many patients in low- and middle-income countries who are sold AMC suspensions would be expected not to keep to the specified storage conditions. We aimed to assess the stability of both ingredients in liquid formulations and dispersible tablets, combined with nationally representative data on access to appropriate storage. Degradation of amoxicillin (AMX) and clavulanic-acid (CLA) was measured in suspensions and dispersible tablets commercially available in Switzerland at different ambient temperatures (8 °C vs. 28 °C over 7 days, and 23 °C vs. 28 °C over 24 h, respectively). Data on access to refrigeration and electricity were assessed from the USAID-funded Demographic and Health Survey program. In suspensions, CLA degraded to a maximum of 12.9% (95% CI −55.7%, +29.9%) at 8°C and 72.3% (95% CI −82.8%, −61.8%) at a 28 °C ambient temperature during an observation period of 7 days. Dispersible tablets were observed during 24 h and CLA degraded to 15.4% (95% CI −51.9%, +21.2%) at 23 °C and 21.7% (−28.2%, −15.1%) at a 28 °C ambient temperature. There is relevant degradation of CLA in suspensions during a 7-day course. To overcome the stability challenges for all active components, durable child-appropriate formulations are needed. Until then, prescribers of AMC suspensions or pharmacists who sell the drug need to create awareness for the importance of proper storage conditions regarding effectiveness of both antibiotics and this recommendation should be reflected in the WHO Essential Medicines List for children.

## 1. Introduction

Understanding and tracking patterns of antibiotic consumption over time and across countries is essential to ensure appropriate access. Young children are of particular interest, as they have a disproportionately high use of antibiotics [1].

A recent study analyzed 2011–2015 trends in the consumption of child-appropriate formulations (CAFs, defined as oral liquid formulations and solid formulations that are primarily dissolved or dispersed or become liquid upon swallowing) for under-fives in 36 low- and middle-income countries (LMICs) and 39 high-income countries (HICs) [1]. Antibiotic sales increasing by 8.6% per year mainly driven by LMICs were observed, with amoxicillin (AMX) with and without clavulanic-acid (AMC) being among the antibiotics with the highest sales volume globally [1]. Both AMX and AMC are listed as Access antibiotics on the 2019 revision of the WHO Essential Medicines List for children, this group representing the core set of antibiotics, which should be consistently available at an appropriate quality, formulation and price [2,3].

CAFs of many antibiotics are limited to dry powder suspensions, some of which have to be stored refrigerated once reconstituted, including AMC, due to stability limitations of the active ingredients (a drug substance is said to be stable when no more than 10% of its original content is degraded during the storage period [4]). However, many consumers cannot keep to the specified storage conditions for different reasons, such as lack of access to a refrigerator or irregular power supply. This raises concerns about the inappropriate use of such formulations, especially in LMICs. Given the high rate of use of oral AMC reported in LMICs, it is necessary to explore the stability of either active substance as it would be stored in real life (i.e., in suspension).

We aimed to assess the stability of both AMX and CLA in co-formulated and non-co-formulated commercially available liquid formulations and dispersible tablets under different storage conditions, combined with nationally representative data on access to appropriate storage conditions in a range of LMICs.

## 2. Results

### 2.1. Stability Data

#### 2.1.1. Amoxicillin-Clavulanic Acid: Co-Formulated Suspensions

**Figure 1:**
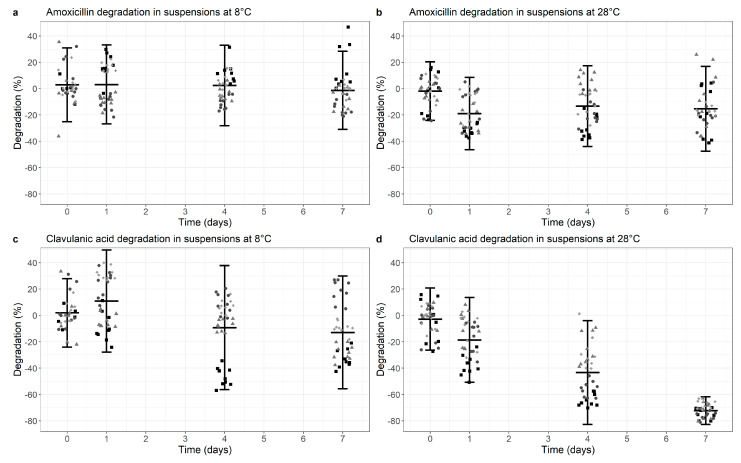
Degradation of Amoxicillin (**a**,**b**) and Clavulanic acid (**c**,**d**) in co-formulated suspensions at 8 °C (**a**,**c**) versus 28 °C (**b**,**d**). Degradation is normalized for the median value at time point 0 (0% degradation). Data points represent the normalized individual measurements for Augmentin Duo (square), Augmentin Trio (circle), Aziclav Duo (triangle) and Aziclav Forte (diamond). Cross bar represents the mean and the whiskers indicate the 95% confidence interval (mean ± 2 SD) per time point.

For AMC co-formulated suspensions, AMX was largely stable after 7 days at 8 °C with a degradation of 1.4% (95% CI −31.0%, +28.3%, *p* = 0.026) (mean of all 4 tested products, Figure 1a). With a higher ambient temperature of 28 °C, AMX degradation increased to 15.3% (95% CI −47.5%, +16.9%, *p* < 0.01) after 7 days (Figure 1b, Table S1 in Supplementary Materials).

The stability data of CLA in co-formulated suspensions at 8 °C and 28 °C is shown in Figure 1c,d and Table S2 in Supplementary Materials. CLA degradation was 12.9% (95% CI −55.7%, +29.9%, *p* < 0.01) at 8 °C and increased to 72.3% (95% CI −82.8%, −61.8%, *p* < 0.01) with a 28 °C ambient temperature.

In summary, there is adequate stability of AMX in co-formulated suspensions under both temperature conditions, but markedly and statistically significant decreased CLA levels from day 4 onwards at an ambient temperature of 28 °C.

#### 2.1.2. Amoxicillin-Clavulanic Acid: Co-Formulated Dispersible Tablets

**Figure 2:**
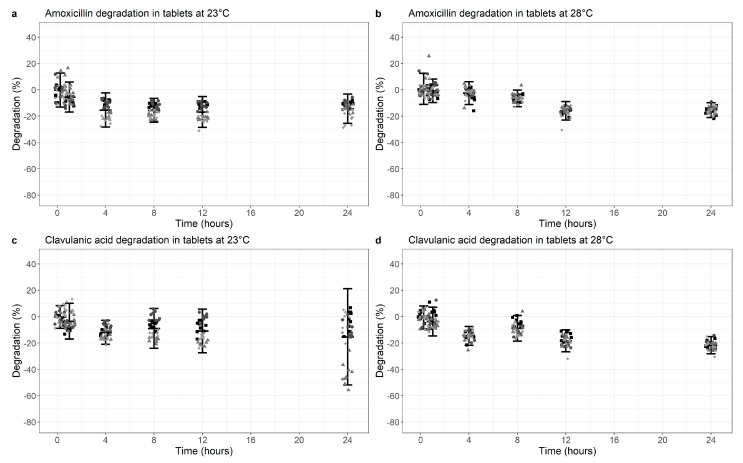
Degradation of Amoxicillin (**a**,**b**) and Clavulanic acid (**c**,**d**) at 23 °C (**a**,**c**) versus 28 °C (**b**,**d**) in co-formulated tablets (dissolved). Degradation is normalized for the median value at time point 0 (0% degradation). Data points represent the normalized individual measurements for Mepha 625 mg (square), Mepha 1000 mg (circle), Sandoz 625 mg (triangle) and Sandoz 1000 mg (diamond) tablets. Cross bar represents the mean and the whiskers indicate the 95% confidence interval (mean ± 2 SD) per time point.

Testing dispersed AMC co-formulated dispersible tablets over a period of maximally 24 h, AMX showed a degradation of 14.4% (95% CI −25.5%, −3.2%, *p* < 0.01) at 23 °C and 15.5% (95% CI −21.1%, −9.9%, *p* < 0.01) at 28 °C (Figure 2a,b, Table S3 in Supplementary Materials).

For the same formulation, CLA degradation showed similar results at 23 °C (mean 15.4%; 95% CI −51.9%, +21.2%, *p* < 0.01) (Figure 2c), but degradation increased to 21.7% (−28.2%, −15.1%, *p* < 0.01) with a 28 °C ambient temperature (Figure 2d, Table S4 in Supplementary Materials).

Overall, some statistically significant degradation was demonstrated for all components, but this is more marked for CLA at 28 °C.

#### 2.1.3. Amoxicillin: Suspensions and Dispersible Tablets

**Figure 3:**
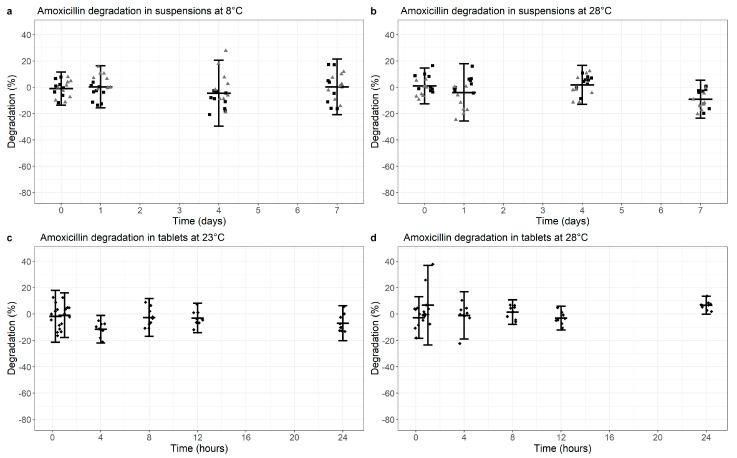
Degradation of Amoxicillin in non-co-formulated suspensions (**a**,**b**) at 8 °C (**a**) versus 28 °C (**b**) or tablets (dissolved; c, d) at 23 °C (**c**) versus 28 °C (**d**). Degradation is normalized for the median value at time point 0 (0% degradation). Data points represent the normalized individual measurements for Mepha (square) and Sandoz (triangle) suspensions and Sandoz 1000 mg tablets (diamonds). Cross bar represents the mean and the whiskers indicate the 95% confidence interval (mean ± 2 SD) per time point.

For non-co-formulated AMX, levels remained stable during a 7-day period at 8 °C (*p* = 0.671) or 28 °C temperature for suspensions with a slightly increased degradation at the higher temperature (9.0%; 95% CI −23.4%, +5.4%, *p* < 0.01), Figure 3a,b, Table S5 in Supplementary Materials).

For dispersible AMX tablets, the amount of AMX remained relatively stable after 24 h at 23 °C and 28 °C (*p* = 0.176 and *p* = 0.007, respectively, Figure 3c,d, Table S6 in Supplementary Materials).

Altogether, AMX was largely stable over 7 days and 24 h in non-co-formulated suspensions and dispersible tablets, respectively, even though the former products were slightly less stable at higher temperatures than the latter.

### 2.2. DHS Data on Access to Electricity and Refrigeration

**Figure 4:**
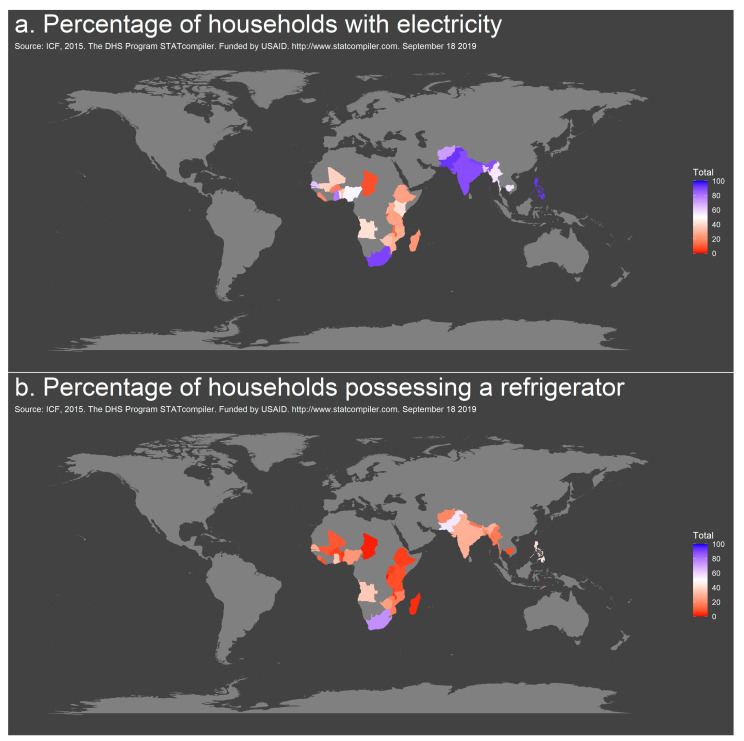
Geographic distribution of access to electricity (**a**) and refrigeration (**b**) in countries of Sub-Saharan Africa and Southeast Asia, 2014–2018.

The number of households with access to electricity ranged from 8.7% in Burundi to 99.8% on the Maldives (mean across 33 countries 44.8%). A minimum of 1.4% of households in Burundi had a refrigerator in the latest survey period, while almost all households (97.8%) had access to refrigeration on the Maldives (mean all countries: 18.5%) [5] (Figure 4a,b, Table S7 in Supplementary Materials). Of note, while ≥75% of households reported access to electricity in Ghana, India, the Maldives, Nepal, Pakistan, the Philippines and South Africa, access to refrigeration was ≥75% only in the Maldives. For 14 countries, average bulb temperature was additionally available and ranged from 15 to 28 °C Table S7 in Supplementary Materials) [5].

## 3. Discussion

This study comprehensively describes the degradation of AMX and CLA as ingredients of co-formulated and non-co-formulated child-appropriate formulations. Our main finding is that CLA in co-formulated suspensions degrades considerably at storage temperatures representative of ambient temperatures in many parts of the world where this antibiotic and formulation is commonly used. While consumption of CAFs of AMX and AMC in HICs have remained largely unchanged, CAF sales increased in LMICs by 6.8% per year [1]. Although the combination is recommended as first-choice treatment only for specific indications [2], LMIC use of AMC almost equaled that of AMX in 2015 [1].

The recommended storage temperature for AMX sodium solution is 2–8 °C. It is stable at higher temperatures for only short periods of time, e.g., 8 or 3 h at 25 °C in sodium chloride or water for injection, respectively [6,7]. There is little data (with robust reporting allowing for reproducibility) on stability at higher temperatures. Stability of at least 90% of AMX at 25 °C and even 40 °C in prepared unit dose syringes for 78 and 10 days, respectively, has been reported for injectable formulations [8]. Recent studies could not confirm these results and demonstrated more rapid degradation under similar conditions. For oral formulations, Mehta et al. describe 90% conservation of reconstituted AMX at 20 °C for only 7 days [9], while Peace et al. observe only 79% conservation at 7 days at a temperature of 27–29 °C [10].

Importantly, CLA is not as heat stable as AMX in either intravenous or reconstituted oral suspension form. Its stability in aqueous solutions has been poorly characterized, though one study reported that after 7 h at 35 °C, about 50% clavulanic acid degraded and 90% after 24 h [11]. Reconstituted oral suspensions appear to be somewhat more stable if storage conditions are appropriate. At 2–8 °C, Mehta et al. observe only 10% degradation after 7 days, while at 20 °C or 27–29 °C ambient temperature, degradation rises to 40% or 45%, respectively [9,10]. Various degradation will likely be the case when oral suspensions are used for administration to young children in settings without secure reliable access to refrigeration. Therefore, it is necessary to explore the stability of either active substance, as it would be stored in real life (i.e., in suspension). Stability testing in this context should reflect how the quality of the active substance varies with time, influenced by a variety of environmental factors such as temperature, humidity and light [10]. In this context, it is important to consider that the content uniformity of a drug may vary according to manufacturer information (i.e., the amount of drug as indicated ±25% according to pharmaceutical industry standards [12]).

In our study, the simulation of inappropriate storage conditions with ambient temperatures of max. 28 °C led to a relevant degradation of CLA in co-formulated suspensions of 72% on average after 7 days, which resembles a regular period of “treatment time” for infections such as community-acquired moderate-severe pneumonia when the reconstituted medication is kept at home [13]. The same but less marked effect was seen for CLA in co-formulated dispersible tablets, if they were dissolved and left for up to 24 h before administering it. However, this scenario is less likely, compared with suspensions being reconstituted once and then stored for the whole treatment period. The most important message for dispersible tablets is that extensive mixing is required before usage in order not to have a precipitate, which then is not ingested.

Our results corroborate earlier studies showing that at 8 °C CLA maintains at least 90% of its initial concentration for 7 days, though at 20 °C only 60% is maintained in the same time period [9] and at 27–29 °C, only 55% [10]. Another study recently showed similar results, with CLA being the stability-limiting factor from day 2 onwards [14]. Additionally, we found that AMX showed somewhat poorer stability in co-formulated products when the product is not stored refrigerated as recommended. Liquid formulations generally tend to have much shorter shelf lives than solid formulations, amongst other reasons because adjuncts such as sweeteners, flavoring as well as suspending, stabilizing and preserving agents are added. These ingredients make the formulations very prone to physical, chemical and microbiological instability [10].

Testing of dispersible tablets shows that both components—AMX and CLA—are satisfactorily stable, even at high ambient temperatures. Unfortunately, these tablets are less flexible in terms of weight-based dosing and the application of technologies used for orally dissolving tablet (ODTs) is limited by the amount of drug that can be incorporated into each unit dose [15]. Therefore, with increasing doses, tablets need to be dissolved in water or any other clean liquid (e.g., breast-milk), which might not be available all the time all over the world. Additionally, dividing a dispersed tablet in several equal portions seems to be impractical due to fast precipitation. However, organizations like UNICEF increasingly supply AMX tablets in a dispersible form as a child-friendly pediatric dosage [16], as they provide various advantages compared with suspensions, including blister packaging simplifying dispensing and supply, tablets offering an accurately measured dose of antibiotic, having a comparatively lower volume and weight, generating less waste and offering greater logistical and supply chain efficiency through to end-users in a community without any cold chain requirements.

In the context of our observations, oral AMC suspensions currently in use are problematic for two main reasons. First, the co-formulated suspension normally is more expensive than AMX alone and is widely sold to a population where a relevant part of households cannot keep to the specified storage conditions. In some of the evaluated countries, the costs for the co-formulated product compared with AMX alone were up to 3 times more expensive: e.g., UNICEF medical supply division: AMX 125 mg per 5 mL, 100 mL suspension: 0.45 USD indicative price versus AMC 125/31.25 mg per 5 mL, 100 mL suspension: 1.41 USD indicative price [17]. For South Africa: AMX 250 mg per 5 mL, 100 mL suspension: R8.27–9.99 versus AMC 250/62.5 mg per 5 mL, 100 mL suspension: R28.92 [18], which is about the same ratio as for Switzerland [19].

Wealth indices are a helpful tool to set the costs per antibiotic course in relation to the income of families in LMICs, when reliable data on income and expenditures are unavailable. Evaluating variables such as access to electricity and refrigeration from the DHS program showed that overall less than 20% of households from the selected countries from the Sub-Saharan and South/Southeast Asian continent possessed a refrigerator and therefore access to proper storage conditions for oral formulations of AMC in 2014–2018. A study from Durban/South Africa (which is listed as an upper-middle income country by the World Bank list of economies, June 2020 [20] and where 75% of households possess a fridge) reports that almost half of the patients stored their antibiotic (AMX) suspensions outside the fridge in common areas of the house like e.g., the kitchen, bedroom or bathroom [21].

Second, there are insufficient data supporting a better efficacy of AMC compared with AMX alone for key indications like community-acquired pneumonia (CAP) in under-fives. Most guidelines (e.g., British Thoracic Society, European Society for Paediatric Infectious Diseases, Infectious Diseases Society of America) recommend oral AMX as first line for CAP and AMC for suspected staphylococcal infection, e.g., in pneumonia associated with influenza [22,23,24,25,26]. There are clear potential benefits of prescribing the combination instead of AMX alone: the patient receives a broader spectrum antibiotic and is covered for a wider range of pathogens. This approach has many advantages, especially in LMIC where access to healthcare is difficult and the patient has no easy possibility to return in case of non-improvement. However, overuse of broader spectrum agents is the main accelerator of emergence of resistance, including resistance to “downstream” antibiotics such as cephalosporins, fluoroquinolones and carbapenems.

Hence, there is urgent need to generate robust trial data to support the use of AMC in specific indications together with a requirement to develop better formulations to overcome the stability challenges for all active components (e.g., dispersible tablets or ODTs), if AMC indeed is more efficacious for the treatment of specific infections. Until these goals are reached, the prescribers of AMC suspensions or pharmacists who sell the drug need to create awareness for the importance of proper storage conditions regarding effectiveness of both antibiotics. If these conditions cannot be reached, i.e., effectiveness of the drug cannot be guaranteed, changing the formulation of AMC (to e.g., solid or dispersible tablets) or changing the drug (to e.g., a second-line therapy) should be considered. This recommendation should be reflected in the WHO Essential Medicines List for children.

## 4. Materials and Methods

### 4.1. Degradation Kinetics of Clavulanic Acid and Amoxicillin

Degradation over time of AMX and AMC suspensions commercially available in Switzerland was tested at different temperatures. Each product was tested in triplicate, three samples were taken from each bottle at every sampling point (i.e., *n* = 3 samples per product and sampling point) and the average degradation was determined during a storage period of 7 days as roughly corresponding to an antibiotic course prescribed in the community. The bottles were stored at 8 °C, corresponding to refrigeration, and at 28 °C, simulating average ambient temperature in many LMICs.

Additionally, stability of commercially available dispersible tablets of AMX and AMC once dispersed according to the manufacturer’s instructions was tested during 24 h at standard room temperature (23 °C) and at 28 °C.

Storage at room temperature was in natural light on the bench in a room with air-conditioning, which kept the temperature at approximately 23 °C all day. The samples stored in the refrigerator were kept in the dark in a temperature-controlled fridge at 8 °C. Storage at elevated temperature was in the dark in a humidity and temperature-controlled incubator at 28 °C. The tested products are listed in Table 1.

All products were prepared according to the manufacturers’ instructions. Suspension bottles were filled to the marked line using tap water and shaken vigorously at reconstitution and before each sample was taken. Dispersible tablets were dissolved in 100 mL tap water and mixed using a magnetic stirrer during sample withdrawal. Constant mixing of the samples during withdrawal was necessary to achieve uniform sampling, since the dispersions separated quickly.

Liquid formulations (suspensions) were diluted 1:1000 with water and dispersions were diluted 1:100. Solvation was assisted by ultrasonic application. To each 100 µL of sample solution 900 µL internal standard solution (1 mg/l AMX-d4 in methanol) were added before measurement. The samples were analyzed using an UltiMate 3000 HPLC system (Thermo Scientific, Reinach, Switzerland) coupled to a triple quadrupole mass spectrometer (TSQ ENDURA, Thermo Scientific, Reinach, Switzerland) applying electrospray ionization as published before [27]. Chromatographic separation was performed on an Accucore™ XL C18 4 µm 150 × 4.6 mm column (Thermo Fisher Scientific, Reinach, Switzerland) with a binary gradient of 10 mM ammonium acetate in water, adjusted to pH 8 with acetic acid (mobile phase A) and 10 mM ammonium acetate and 0.1% formic acid in methanol/acetonitrile (1/1, *v*/*v*) (mobile phase B). Samples were quantified against calibrators containing AMX and CLA in water.

### 4.2. Data Analysis and Visualization

As for each product, triplicate measurements of three samples were taken, *n* = 9 samples were included per product. For the co-formulated suspensions and tablets, four products were analyzed, leading to a total of *n* = 36 samples available for statistical analysis. Two non-co-formulated suspensions and one non-co-formulated tablet were included in the analysis, which resulted in a total of *n* = 18 and *n* = 9 samples available for statistical analysis, respectively. A paired t-test was used to compare the means of degradation across all formulations (*n* = 36, *n* = 18, or *n* = 9 as described above) at different time points with the mean of the first measurements, with a calculated p-value < 0.01 considered to be statistically significantly different.

Additionally, for each time point, descriptive statistics were applied and the mean, median, standard deviation (SD), standard error and 95% confidence interval (95% CI, mean ± 2SD) of triplicates of all three samples from bottles or tablet dispersions were calculated. Data was normalized by the median per product, with 0% degradation at the start of the study. Data visualization of the individual and mean ± 2 SD measurements per time point was performed using R (version 3.5.1) and RStudio (version 1.1.456).

### 4.3. DHS Data on Access to Electricity and Refrigeration

To assess the likely frequency of a lack of cold storage in the home, the STATcompiler from the USAID-funded Demographic and Health Survey (DHS) program (www.statcompiler.com, accessed on 27 October 2020) was used to assess datasets for access to refrigeration and electricity for 33 countries from Sub-Saharan Africa and South/Southeast Asia [5]. Surveys were run within the last five years with available data from 2014–2018. Map visualizations were created using the *ggmap* and *maps* packages in R.

## Figures and Tables

**Table 1:** Tested commercially available co-formulated and non-co-formulated products.

Manufacturer	Product
Spirig HealthCare (Egerkingen, Switzerland)	Aziclav Duo (400 mg, 57 mg/mL) Aziclav Forte (250 mg, 62.5 mg/5mL)
GlaxoSmithKline (Brentford, United Kingdom)	Augmentin Duo (400 mg, 57 mg/5mL) Augmentin Trio Forte (250 mg, 62.5 mg/5mL)
Mepha Pharma (Basel, Switzerland)	Amoxi-Mepha (200 mg/4mL) Co-Amoxi-Mepha Dispersible 1000 mg (875 mg/125 mg) Co-Amoxi-Mepha Dispersible 625 mg (500 mg/125 mg)
Sandoz Pharmaceuticals (Rotkreuz, Switzerland)	Amoxicillin Sandoz (200 mg Amoxicillin/4 mL) Amoxicillin Sandoz 1000 (1000 mg Amoxicillin per tablet) Co-Amoxicillin Sandoz 625 (500 mg/125 mg per tablet) Co-Amoxicillin Sandoz 1 g (875 mg/125 mg per tablet)

## Data Availability

The data represented in this study are available in the article or uploaded as supplementary information.

## Supplementary Materials

The following are available online at https://www.mdpi.com/2079-6382/10/2/225/s1, Table S1: Degradation of Amoxicillin in the Amoxicillin-clavulanic acid co-formulated suspensions at 28 °C and 8 °C; Table S2: Degradation of Clavulanic acid in Amoxicillin-clavulanic acid co-formulated suspensions at 28 °C and 8 °C; Table S3: Degradation of Amoxicillin in dispersed Amoxicillin-clavulanic acid co-formulated dispersible tablets at 28 °C and 23 °C; Table S4: Degradation of Clavulanic acid in dispersed Amoxicillin-clavulanic acid co-formulated dispersible tablets at 28 °C and 23 °C; Table S5: Degradation of Amoxicillin in non-co-formulated suspensions at 28 °C and 8 °C; Table S6: Degradation of Amoxicillin in non-co-formulated dispersible tablets at 28 °C and 23 °C; Table S7: Percentage (%) of households in represented African and Asian countries having access to electricity or a refrigerator, assessed during DHS or MIS surveys from 2014–2018.
