# Supplementary material for: Insufficient Stability of Clavulanic Acid in Widely Used Child-Appropriate Formulations
Source: Antibiotics (Basel). 2021 Feb 23;10(2):225. doi: 10.3390/antibiotics10020225 (PMC7927114; doi:10.3390/antibiotics10020225)
Supplement: Supplementary file 1 [file antibiotics-10-00225-s001.zip › Amox Co-amox stability_Mack_Table S1.docx]

**Table 1.** Degradation of Amoxicillin in the Amoxicillin-clavulanic acid co-formulated suspensions at 28°C and 8°C. Mean, median, standard deviation (sd), standard error (se), and 95% confidence interval (“lower” and “upper”) are reported for each of the four tested products (Augmentin Duo/Trio, and Aziclav Duo/Forte).

| **days** | **temp** | **type** | **N** | **mean** | **median** | **sd** | **se** | **lower** | **upper** |
| --- | --- | --- | --- | --- | --- | --- | --- | --- | --- |
| 0 | 28°C | Aug Duo | 9 | -2.39 | 0.00 | 15.54 | 5.18 | -33.47 | 28.69 |
| 0 | 28°C | Aug Trio | 9 | -4.65 | 0.00 | 13.37 | 4.46 | -31.40 | 22.09 |
| 0 | 28°C | Azi Duo | 9 | -1.39 | 0.00 | 6.50 | 2.17 | -14.39 | 11.61 |
| 0 | 28°C | Azi Forte | 9 | 0.88 | 0.00 | 7.77 | 2.59 | -14.66 | 16.42 |
| 0 | 28°C | all | 36 | -1.89 | 0.00 | 11.12 | 1.85 | -24.12 | 20.35 |
| 1 | 28°C | Aug Duo | 9 | -31.76 | -32.36 | 4.07 | 1.36 | -39.91 | -23.61 |
| 1 | 28°C | Aug Trio | 9 | -10.66 | -4.67 | 14.24 | 4.75 | -39.13 | 17.82 |
| 1 | 28°C | Azi Duo | 9 | -25.20 | -26.46 | 8.32 | 2.77 | -41.84 | -8.57 |
| 1 | 28°C | Azi Forte | 9 | -8.09 | -4.05 | 9.83 | 3.28 | -27.75 | 11.57 |
| 1 | 28°C | all | 36 | -18.93 | -23.93 | 13.72 | 2.29 | -46.37 | 8.52 |
| 4 | 28°C | Aug Duo | 9 | -29.73 | -32.36 | 8.74 | 2.91 | -47.21 | -12.25 |
| 4 | 28°C | Aug Trio | 9 | -18.41 | -22.07 | 9.14 | 3.05 | -36.70 | -0.12 |
| 4 | 28°C | Azi Duo | 9 | 5.60 | 6.52 | 6.64 | 2.21 | -7.68 | 18.87 |
| 4 | 28°C | Azi Forte | 9 | -10.89 | -5.01 | 9.13 | 3.04 | -29.16 | 7.38 |
| 4 | 28°C | all | 36 | -13.36 | -14.21 | 15.33 | 2.56 | -44.03 | 17.31 |
| 7 | 28°C | Aug Duo | 9 | -16.83 | -18.86 | 19.46 | 6.49 | -55.75 | 22.09 |
| 7 | 28°C | Aug Trio | 9 | -19.36 | -20.71 | 10.62 | 3.54 | -40.60 | 1.88 |
| 7 | 28°C | Azi Duo | 9 | -8.50 | -9.29 | 23.65 | 7.88 | -55.80 | 38.79 |
| 7 | 28°C | Azi Forte | 9 | -16.53 | -16.97 | 3.20 | 1.07 | -22.92 | -10.14 |
| 7 | 28°C | all | 36 | -15.31 | -17.59 | 16.11 | 2.69 | -47.53 | 16.92 |
| **days** | **temp** | **type** | **N** | **mean** | **median** | **sd** | **se** | **lower** | **upper** |
| 0 | 8°C | Aug Duo | 6 | -0.58 | 0.00 | 7.45 | 3.04 | -15.48 | 14.32 |
| 0 | 8°C | Aug Trio | 9 | 8.17 | 0.00 | 14.26 | 4.75 | -20.36 | 36.70 |
| 0 | 8°C | Azi Duo | 8 | -0.83 | 0.00 | 19.82 | 7.01 | -40.46 | 38.80 |
| 0 | 8°C | Azi Forte | 8 | 3.14 | 0.00 | 10.83 | 3.83 | -18.53 | 24.80 |
| 0 | 8°C | all | 31 | 2.86 | 0.00 | 14.04 | 2.52 | -25.21 | 30.93 |
| 1 | 8°C | Aug Duo | 9 | 17.05 | 15.35 | 9.81 | 3.27 | -2.56 | 36.66 |
| 1 | 8°C | Aug Trio | 9 | -11.55 | -12.45 | 5.82 | 1.94 | -23.19 | 0.09 |
| 1 | 8°C | Azi Duo | 9 | -9.18 | -8.42 | 4.50 | 1.50 | -18.18 | -0.17 |
| 1 | 8°C | Azi Forte | 9 | 16.18 | 14.68 | 3.39 | 1.13 | 9.40 | 22.97 |
| 1 | 8°C | all | 36 | 3.13 | -3.18 | 15.00 | 2.50 | -26.88 | 33.13 |
| 4 | 8°C | Aug Duo | 9 | 18.44 | 11.68 | 19.51 | 6.50 | -20.59 | 57.47 |
| 4 | 8°C | Aug Trio | 9 | -11.26 | -12.34 | 4.77 | 1.59 | -20.80 | -1.73 |
| 4 | 8°C | Azi Duo | 9 | -3.22 | -5.25 | 5.31 | 1.77 | -13.85 | 7.41 |
| 4 | 8°C | Azi Forte | 9 | 5.45 | 3.93 | 6.92 | 2.31 | -8.39 | 19.28 |
| 4 | 8°C | all | 36 | 2.35 | 0.97 | 15.31 | 2.55 | -28.26 | 32.96 |
| 7 | 8°C | Aug Duo | 9 | 16.04 | 7.08 | 16.73 | 5.58 | -17.41 | 49.50 |
| 7 | 8°C | Aug Trio | 9 | -14.79 | -17.78 | 5.75 | 1.92 | -26.28 | -3.29 |
| 7 | 8°C | Azi Duo | 9 | -6.50 | -3.85 | 7.52 | 2.51 | -21.53 | 8.54 |
| 7 | 8°C | Azi Forte | 9 | -0.18 | 0.10 | 4.33 | 1.44 | -8.83 | 8.48 |
| 7 | 8°C | all | 36 | -1.35 | -0.50 | 14.84 | 2.47 | -31.03 | 28.32 |
