# Supplementary material for: Insufficient Stability of Clavulanic Acid in Widely Used Child-Appropriate Formulations
Source: Antibiotics (Basel). 2021 Feb 23;10(2):225. doi: 10.3390/antibiotics10020225 (PMC7927114; doi:10.3390/antibiotics10020225)
Supplement: Supplementary file 1 [file antibiotics-10-00225-s001.zip › Amox Co-amox stability_Mack_Table S2.docx]

**Table S2.** Degradation of Clavulanic acid in Amoxicillin-clavulanic acid co-formulated suspensions at 28°C and 8°C. Mean, median, standard deviation (sd), standard error (se), and 95% confidence interval (“lower” and “upper”) are reported for each of the four tested products (Augmentin Duo/Trio, and Aziclav Duo/Forte).

| **days** | **temp** | **type** | **N** | **mean** | **median** | **sd** | **se** | **lower** | **upper** |
| --- | --- | --- | --- | --- | --- | --- | --- | --- | --- |
| 0 | 28°C | Aug Duo | 9 | -3.43 | 0.00 | 16.31 | 5.44 | -36.04 | 29.18 |
| 0 | 28°C | Aug Trio | 9 | -6.61 | 0.00 | 13.59 | 4.53 | -33.78 | 20.57 |
| 0 | 28°C | Azi Duo | 9 | 0.18 | 0.00 | 7.51 | 2.50 | -14.84 | 15.20 |
| 0 | 28°C | Azi Forte | 9 | -1.21 | 0.00 | 8.32 | 2.77 | -17.84 | 15.43 |
| 0 | 28°C | all | 36 | -2.77 | 0.00 | 11.77 | 1.96 | -26.30 | 20.77 |
| 1 | 28°C | Aug Duo | 9 | -38.24 | -40.53 | 8.20 | 2.73 | -54.64 | -21.85 |
| 1 | 28°C | Aug Trio | 9 | -15.82 | -8.95 | 11.24 | 3.75 | -38.29 | 6.65 |
| 1 | 28°C | Azi Duo | 9 | -9.93 | -11.68 | 13.03 | 4.34 | -35.99 | 16.13 |
| 1 | 28°C | Azi Forte | 9 | -10.38 | -4.76 | 12.74 | 4.25 | -35.86 | 15.09 |
| 1 | 28°C | all | 36 | -18.59 | -17.44 | 16.06 | 2.68 | -50.72 | 13.53 |
| 4 | 28°C | Aug Duo | 9 | -64.39 | -66.41 | 4.84 | 1.61 | -74.08 | -54.71 |
| 4 | 28°C | Aug Trio | 9 | -55.79 | -54.87 | 5.97 | 1.99 | -67.74 | -43.84 |
| 4 | 28°C | Azi Duo | 9 | -25.96 | -31.15 | 12.00 | 4.00 | -49.95 | -1.96 |
| 4 | 28°C | Azi Forte | 9 | -27.24 | -31.21 | 13.86 | 4.62 | -54.95 | 0.48 |
| 4 | 28°C | all | 36 | -43.34 | -43.02 | 19.71 | 3.29 | -82.77 | -3.92 |
| 7 | 28°C | Aug Duo | 9 | -74.77 | -75.27 | 4.07 | 1.36 | -82.90 | -66.63 |
| 7 | 28°C | Aug Trio | 9 | -75.45 | -74.74 | 3.15 | 1.05 | -81.75 | -69.16 |
| 7 | 28°C | Azi Duo | 9 | -71.57 | -70.66 | 6.24 | 2.08 | -84.05 | -59.10 |
| 7 | 28°C | Azi Forte | 9 | -67.43 | -66.31 | 3.01 | 1.00 | -73.46 | -61.41 |
| 7 | 28°C | all | 36 | -72.31 | -71.57 | 5.23 | 0.87 | -82.77 | -61.84 |
| **days** | **temp** | **type** | **N** | **mean** | **median** | **sd** | **se** | **lower** | **upper** |
| 0 | 8°C | Aug Duo | 6 | -0.51 | 0.00 | 6.81 | 2.78 | -14.12 | 13.11 |
| 0 | 8°C | Aug Trio | 9 | 5.75 | 0.00 | 15.70 | 5.23 | -25.65 | 37.16 |
| 0 | 8°C | Azi Duo | 8 | -1.09 | 0.00 | 17.39 | 6.15 | -35.86 | 33.69 |
| 0 | 8°C | Azi Forte | 8 | 2.32 | 0.00 | 8.29 | 2.93 | -14.27 | 18.90 |
| 0 | 8°C | all | 31 | 1.89 | 0.00 | 12.97 | 2.33 | -24.04 | 27.82 |
| 1 | 8°C | Aug Duo | 9 | -8.89 | -11.33 | 11.11 | 3.70 | -31.11 | 13.33 |
| 1 | 8°C | Aug Trio | 9 | 24.17 | 26.61 | 9.96 | 3.32 | 4.25 | 44.09 |
| 1 | 8°C | Azi Duo | 9 | -3.69 | -7.04 | 5.37 | 1.79 | -14.43 | 7.05 |
| 1 | 8°C | Azi Forte | 9 | 31.87 | 30.69 | 4.69 | 1.56 | 22.50 | 41.24 |
| 1 | 8°C | all | 36 | 10.87 | 9.31 | 19.40 | 3.23 | -27.93 | 49.66 |
| 4 | 8°C | Aug Duo | 9 | -46.47 | -48.05 | 7.27 | 2.42 | -61.02 | -31.93 |
| 4 | 8°C | Aug Trio | 9 | 9.49 | 8.34 | 8.20 | 2.73 | -6.91 | 25.89 |
| 4 | 8°C | Azi Duo | 9 | -7.68 | -7.59 | 4.33 | 1.44 | -16.33 | 0.98 |
| 4 | 8°C | Azi Forte | 9 | 7.35 | 7.19 | 4.87 | 1.62 | -2.38 | 17.08 |
| 4 | 8°C | all | 36 | -9.33 | -2.21 | 23.56 | 3.93 | -56.45 | 37.80 |
| 7 | 8°C | Aug Duo | 9 | -32.88 | -35.21 | 7.09 | 2.36 | -47.06 | -18.71 |
| 7 | 8°C | Aug Trio | 9 | 18.38 | 19.18 | 8.45 | 2.82 | 1.48 | 35.27 |
| 7 | 8°C | Azi Duo | 9 | -28.00 | -28.22 | 5.99 | 2.00 | -39.98 | -16.01 |
| 7 | 8°C | Azi Forte | 9 | -9.06 | -9.53 | 4.49 | 1.50 | -18.04 | -0.07 |
| 7 | 8°C | all | 36 | -12.89 | -18.98 | 21.38 | 3.56 | -55.66 | 29.88 |
