# Supplementary material for: Insufficient Stability of Clavulanic Acid in Widely Used Child-Appropriate Formulations
Source: Antibiotics (Basel). 2021 Feb 23;10(2):225. doi: 10.3390/antibiotics10020225 (PMC7927114; doi:10.3390/antibiotics10020225)
Supplement: Supplementary file 1 [file antibiotics-10-00225-s001.zip › Amox Co-amox stability_Mack_Table S3.docx]

**Table S3.** Degradation of Amoxicillin in dispersed Amoxicillin-clavulanic acid co-formulated dispersible tablets at 28°C and 23°C. Mean, median, standard deviation (sd), standard error (se), and 95% confidence inTable 625. and 1000 mg).

| **hours** | **temp** | **type** | **N** | **mean** | **median** | **sd** | **se** | **lower** | **upper** |
| --- | --- | --- | --- | --- | --- | --- | --- | --- | --- |
| 0.25 | 28°C | M-625 | 9 | 0.11 | 0.00 | 2.43 | 0.81 | -4.75 | 4.97 |
| 0.25 | 28°C | M-1000 | 9 | 0.55 | 0.00 | 6.21 | 2.07 | -11.87 | 12.97 |
| 0.25 | 28°C | S-625 | 9 | 3.26 | 0.00 | 9.13 | 3.04 | -15.00 | 21.53 |
| 0.25 | 28°C | S-1000 | 9 | -1.04 | 0.00 | 3.77 | 1.26 | -8.58 | 6.51 |
| 0.25 | 28°C | all | 36 | 0.72 | 0.00 | 5.92 | 0.99 | -11.12 | 12.56 |
| 1 | 28°C | M-625 | 9 | -1.98 | -1.89 | 3.72 | 1.24 | -9.42 | 5.47 |
| 1 | 28°C | M-1000 | 9 | 2.61 | 3.25 | 4.82 | 1.61 | -7.03 | 12.25 |
| 1 | 28°C | S-625 | 9 | -3.01 | -3.28 | 2.39 | 0.80 | -7.78 | 1.77 |
| 1 | 28°C | S-1000 | 9 | -0.65 | -2.28 | 4.76 | 1.59 | -10.18 | 8.88 |
| 1 | 28°C | all | 36 | -0.76 | -1.68 | 4.42 | 0.74 | -9.60 | 8.09 |
| 4 | 28°C | M-625 | 9 | -5.01 | -5.29 | 5.16 | 1.72 | -15.32 | 5.30 |
| 4 | 28°C | M-1000 | 9 | -1.38 | -0.68 | 1.90 | 0.63 | -5.19 | 2.42 |
| 4 | 28°C | S-625 | 9 | -2.00 | -1.26 | 5.56 | 1.85 | -13.13 | 9.13 |
| 4 | 28°C | S-1000 | 9 | -1.87 | -1.46 | 3.51 | 1.17 | -8.90 | 5.15 |
| 4 | 28°C | all | 36 | -2.57 | -1.47 | 4.35 | 0.72 | -11.26 | 6.13 |
| 8 | 28°C | M-625 | 9 | -6.67 | -6.55 | 3.00 | 1.00 | -12.66 | -0.68 |
| 8 | 28°C | M-1000 | 9 | -5.60 | -5.59 | 1.84 | 0.61 | -9.28 | -1.92 |
| 8 | 28°C | S-625 | 9 | -5.21 | -5.84 | 3.95 | 1.32 | -13.11 | 2.68 |
| 8 | 28°C | S-1000 | 9 | -8.81 | -9.16 | 2.62 | 0.87 | -14.06 | -3.56 |
| 8 | 28°C | all | 36 | -6.57 | -6.05 | 3.16 | 0.53 | -12.89 | -0.26 |
| 12 | 28°C | M-625 | 9 | -15.60 | -14.76 | 2.90 | 0.97 | -21.41 | -9.80 |
| 12 | 28°C | M-1000 | 9 | -15.31 | -14.85 | 1.40 | 0.47 | -18.10 | -12.51 |
| 12 | 28°C | S-625 | 9 | -15.03 | -14.64 | 1.64 | 0.55 | -18.31 | -11.76 |
| 12 | 28°C | S-1000 | 9 | -18.04 | -18.57 | 5.83 | 1.94 | -29.69 | -6.39 |
| 12 | 28°C | all | 36 | -16.00 | -14.84 | 3.50 | 0.58 | -22.99 | -9.00 |
| 24 | 28°C | M-625 | 9 | -16.80 | -16.69 | 2.87 | 0.96 | -22.54 | -11.06 |
| 24 | 28°C | M-1000 | 9 | -14.10 | -13.88 | 2.54 | 0.85 | -19.18 | -9.03 |
| 24 | 28°C | S-625 | 9 | -15.47 | -15.46 | 2.28 | 0.76 | -20.03 | -10.90 |
| 24 | 28°C | S-1000 | 9 | -15.66 | -15.90 | 3.16 | 1.05 | -21.99 | -9.34 |
| 24 | 28°C | all | 36 | -15.51 | -15.39 | 2.79 | 0.46 | -21.09 | -9.93 |
| **hours** | **temp** | **type** | **N** | **mean** | **median** | **sd** | **se** | **lower** | **upper** |
| 0.25 | 23°C | M-625 | 9 | -1.30 | 0.00 | 4.18 | 1.39 | -9.67 | 7.07 |
| 0.25 | 23°C | M-1000 | 9 | -1.42 | 0.00 | 7.05 | 2.35 | -15.52 | 12.67 |
| 0.25 | 23°C | S-625 | 9 | 3.35 | 0.00 | 6.71 | 2.24 | -10.06 | 16.77 |
| 0.25 | 23°C | S-1000 | 9 | -1.51 | 0.00 | 7.13 | 2.38 | -15.77 | 12.76 |
| 0.25 | 23°C | all | 36 | -0.22 | 0.00 | 6.45 | 1.08 | -13.13 | 12.69 |
| 1 | 23°C | M-625 | 9 | -8.27 | -8.30 | 2.02 | 0.67 | -12.31 | -4.22 |
| 1 | 23°C | M-1000 | 9 | -9.04 | -8.30 | 3.09 | 1.03 | -15.23 | -2.86 |
| 1 | 23°C | S-625 | 9 | -0.81 | -2.46 | 7.62 | 2.54 | -16.04 | 14.42 |
| 1 | 23°C | S-1000 | 9 | -3.92 | -3.32 | 4.41 | 1.47 | -12.74 | 4.90 |
| 1 | 23°C | all | 36 | -5.51 | -6.85 | 5.69 | 0.95 | -16.88 | 5.86 |
| 4 | 23°C | M-625 | 9 | -8.66 | -8.07 | 1.84 | 0.61 | -12.34 | -4.98 |
| 4 | 23°C | M-1000 | 9 | -10.42 | -9.81 | 2.68 | 0.89 | -15.78 | -5.06 |
| 4 | 23°C | S-625 | 9 | -19.43 | -20.24 | 2.19 | 0.73 | -23.81 | -15.04 |
| 4 | 23°C | S-1000 | 9 | -22.89 | -22.56 | 2.46 | 0.82 | -27.80 | -17.97 |
| 4 | 23°C | all | 36 | -15.35 | -15.23 | 6.45 | 1.07 | -28.24 | -2.46 |
| 8 | 23°C | M-625 | 9 | -12.32 | -11.84 | 1.99 | 0.66 | -16.30 | -8.33 |
| 8 | 23°C | M-1000 | 9 | -11.40 | -11.07 | 2.55 | 0.85 | -16.50 | -6.30 |
| 8 | 23°C | S-625 | 9 | -20.01 | -19.68 | 2.78 | 0.93 | -25.58 | -14.45 |
| 8 | 23°C | S-1000 | 9 | -18.57 | -18.79 | 2.43 | 0.81 | -23.42 | -13.72 |
| 8 | 23°C | all | 36 | -15.57 | -15.48 | 4.48 | 0.75 | -24.54 | -6.61 |
| 12 | 23°C | M-625 | 9 | -11.28 | -11.27 | 1.66 | 0.55 | -14.60 | -7.97 |
| 12 | 23°C | M-1000 | 9 | -11.95 | -12.18 | 2.59 | 0.86 | -17.12 | -6.78 |
| 12 | 23°C | S-625 | 9 | -21.06 | -21.52 | 1.87 | 0.62 | -24.80 | -17.32 |
| 12 | 23°C | S-1000 | 9 | -23.14 | -22.84 | 3.38 | 1.13 | -29.89 | -16.39 |
| 12 | 23°C | all | 36 | -16.86 | -16.59 | 5.87 | 0.98 | -28.60 | -5.12 |
| 24 | 23°C | M-625 | 9 | -10.65 | -10.61 | 1.55 | 0.52 | -13.74 | -7.55 |
| 24 | 23°C | M-1000 | 9 | -10.83 | -10.20 | 3.39 | 1.13 | -17.61 | -4.06 |
| 24 | 23°C | S-625 | 9 | -14.27 | -14.08 | 2.60 | 0.87 | -19.47 | -9.06 |
| 24 | 23°C | S-1000 | 9 | -21.66 | -21.20 | 5.12 | 1.71 | -31.89 | -11.42 |
| 24 | 23°C | all | 36 | -14.35 | -13.55 | 5.58 | 0.93 | -25.51 | -3.19 |
