# Supplementary material for: Insufficient Stability of Clavulanic Acid in Widely Used Child-Appropriate Formulations
Source: Antibiotics (Basel). 2021 Feb 23;10(2):225. doi: 10.3390/antibiotics10020225 (PMC7927114; doi:10.3390/antibiotics10020225)
Supplement: Supplementary file 1 [file antibiotics-10-00225-s001.zip › Amox Co-amox stability_Mack_Table S4.docx]

**Table 4.** Degradation of Clavulanic acid in dispersed Amoxicillin-clavulanic acid co-formulated dispersible tablets at 28°C and 23°C. Mean, median, standard deviation (sd), standard error (se), and 95% confidence interval (“lower” and “upper”) are reported for each of the four tested products (Mepha and Sandoz, each 625 and 1000 mg).

| **hours** | **temp** | **type** | **N** | **mean** | **median** | **sd** | **se** | **lower** | **upper** |
| --- | --- | --- | --- | --- | --- | --- | --- | --- | --- |
| 0.25 | 28°C | M-625 | 9 | 0.48 | 0.00 | 2.97 | 0.99 | -5.46 | 6.42 |
| 0.25 | 28°C | M-1000 | 9 | -1.74 | 0.00 | 3.00 | 1.00 | -7.74 | 4.27 |
| 0.25 | 28°C | S-625 | 9 | -1.13 | 0.00 | 5.65 | 1.88 | -12.43 | 10.18 |
| 0.25 | 28°C | S-1000 | 9 | -0.06 | 0.00 | 5.41 | 1.80 | -10.88 | 10.77 |
| 0.25 | 28°C | all | 36 | -0.61 | 0.00 | 4.34 | 0.72 | -9.29 | 8.07 |
| 1 | 28°C | M-625 | 9 | 0.90 | 0.90 | 5.68 | 1.89 | -10.47 | 12.27 |
| 1 | 28°C | M-1000 | 9 | -4.54 | -6.30 | 6.65 | 2.22 | -17.84 | 8.75 |
| 1 | 28°C | S-625 | 9 | -6.05 | -7.02 | 2.67 | 0.89 | -11.39 | -0.70 |
| 1 | 28°C | S-1000 | 9 | -5.59 | -4.40 | 3.51 | 1.17 | -12.62 | 1.44 |
| 1 | 28°C | all | 36 | -3.82 | -4.68 | 5.47 | 0.91 | -14.75 | 7.11 |
| 4 | 28°C | M-625 | 9 | -14.09 | -14.24 | 3.48 | 1.16 | -21.05 | -7.13 |
| 4 | 28°C | M-1000 | 9 | -15.33 | -15.34 | 2.69 | 0.90 | -20.70 | -9.95 |
| 4 | 28°C | S-625 | 9 | -15.27 | -14.30 | 4.48 | 1.49 | -24.24 | -6.30 |
| 4 | 28°C | S-1000 | 9 | -13.55 | -13.41 | 3.65 | 1.22 | -20.85 | -6.25 |
| 4 | 28°C | all | 36 | -14.56 | -14.27 | 3.56 | 0.59 | -21.68 | -7.44 |
| 8 | 28°C | M-625 | 9 | -4.46 | -5.47 | 3.22 | 1.07 | -10.89 | 1.97 |
| 8 | 28°C | M-1000 | 9 | -10.85 | -11.18 | 3.11 | 1.04 | -17.08 | -4.62 |
| 8 | 28°C | S-625 | 9 | -8.72 | -9.25 | 5.81 | 1.94 | -20.35 | 2.91 |
| 8 | 28°C | S-1000 | 9 | -11.32 | -11.84 | 4.11 | 1.37 | -19.55 | -3.10 |
| 8 | 28°C | all | 36 | -8.84 | -8.77 | 4.87 | 0.81 | -18.58 | 0.91 |
| 12 | 28°C | M-625 | 9 | -15.23 | -14.80 | 3.40 | 1.13 | -22.02 | -8.43 |
| 12 | 28°C | M-1000 | 9 | -20.14 | -21.59 | 3.20 | 1.07 | -26.54 | -13.74 |
| 12 | 28°C | S-625 | 9 | -18.20 | -17.91 | 3.12 | 1.04 | -24.44 | -11.97 |
| 12 | 28°C | S-1000 | 9 | -19.78 | -18.27 | 5.23 | 1.74 | -30.23 | -9.32 |
| 12 | 28°C | all | 36 | -18.34 | -18.23 | 4.16 | 0.69 | -26.66 | -10.02 |
| 24 | 28°C | M-625 | 9 | -20.90 | -21.29 | 2.51 | 0.84 | -25.93 | -15.88 |
| 24 | 28°C | M-1000 | 9 | -20.84 | -20.50 | 3.46 | 1.15 | -27.77 | -13.92 |
| 24 | 28°C | S-625 | 9 | -22.20 | -20.83 | 2.72 | 0.91 | -27.65 | -16.75 |
| 24 | 28°C | S-1000 | 9 | -22.80 | -22.59 | 4.24 | 1.41 | -31.28 | -14.31 |
| 24 | 28°C | all | 36 | -21.68 | -21.37 | 3.27 | 0.55 | -28.23 | -15.14 |
| **hours** | **temp** | **type** | **N** | **mean** | **median** | **sd** | **se** | **lower** | **upper** |
| 0.25 | 23°C | M-625 | 9 | -1.34 | 0.00 | 2.52 | 0.84 | -6.38 | 3.70 |
| 0.25 | 23°C | M-1000 | 9 | 0.18 | 0.00 | 4.56 | 1.52 | -8.95 | 9.31 |
| 0.25 | 23°C | S-625 | 9 | -0.76 | 0.00 | 4.60 | 1.53 | -9.97 | 8.45 |
| 0.25 | 23°C | S-1000 | 9 | 0.79 | 0.00 | 5.38 | 1.79 | -9.97 | 11.55 |
| 0.25 | 23°C | all | 36 | -0.28 | 0.00 | 4.29 | 0.71 | -8.86 | 8.29 |
| 1 | 23°C | M-625 | 9 | -9.16 | -9.32 | 2.17 | 0.72 | -13.49 | -4.83 |
| 1 | 23°C | M-1000 | 9 | -6.22 | -6.48 | 1.76 | 0.59 | -9.74 | -2.70 |
| 1 | 23°C | S-625 | 9 | -4.43 | -5.28 | 4.57 | 1.52 | -13.57 | 4.71 |
| 1 | 23°C | S-1000 | 9 | 5.69 | 4.91 | 5.53 | 1.84 | -5.37 | 16.76 |
| 1 | 23°C | all | 36 | -3.53 | -6.17 | 6.76 | 1.13 | -17.04 | 9.98 |
| 4 | 23°C | M-625 | 9 | -11.88 | -11.66 | 2.04 | 0.68 | -15.96 | -7.79 |
| 4 | 23°C | M-1000 | 9 | -6.54 | -6.43 | 2.53 | 0.84 | -11.59 | -1.48 |
| 4 | 23°C | S-625 | 9 | -14.76 | -15.07 | 2.73 | 0.91 | -20.21 | -9.30 |
| 4 | 23°C | S-1000 | 9 | -14.46 | -16.30 | 4.87 | 1.62 | -24.19 | -4.73 |
| 4 | 23°C | all | 36 | -11.91 | -12.19 | 4.55 | 0.76 | -21.00 | -2.81 |
| 8 | 23°C | M-625 | 9 | -6.28 | -6.17 | 2.50 | 0.83 | -11.28 | -1.28 |
| 8 | 23°C | M-1000 | 9 | 1.08 | 1.56 | 3.51 | 1.17 | -5.93 | 8.09 |
| 8 | 23°C | S-625 | 9 | -17.51 | -17.00 | 1.72 | 0.57 | -20.94 | -14.08 |
| 8 | 23°C | S-1000 | 9 | -13.09 | -12.56 | 2.01 | 0.67 | -17.11 | -9.06 |
| 8 | 23°C | all | 36 | -8.95 | -10.56 | 7.54 | 1.26 | -24.02 | 6.12 |
| 12 | 23°C | M-625 | 9 | -7.85 | -6.67 | 4.67 | 1.56 | -17.19 | 1.48 |
| 12 | 23°C | M-1000 | 9 | -0.17 | -0.01 | 2.42 | 0.81 | -5.01 | 4.66 |
| 12 | 23°C | S-625 | 9 | -19.65 | -19.60 | 2.49 | 0.83 | -24.63 | -14.68 |
| 12 | 23°C | S-1000 | 9 | -16.01 | -15.32 | 3.35 | 1.12 | -22.70 | -9.32 |
| 12 | 23°C | all | 36 | -10.92 | -12.91 | 8.29 | 1.38 | -27.50 | 5.65 |
| 24 | 23°C | M-625 | 9 | -2.62 | -1.89 | 7.35 | 2.45 | -17.32 | 12.08 |
| 24 | 23°C | M-1000 | 9 | -9.66 | -12.20 | 6.14 | 2.05 | -21.94 | 2.62 |
| 24 | 23°C | S-625 | 9 | -43.55 | -45.97 | 8.78 | 2.93 | -61.10 | -25.99 |
| 24 | 23°C | S-1000 | 9 | -5.62 | -5.54 | 8.41 | 2.80 | -22.43 | 11.20 |
| 24 | 23°C | all | 36 | -15.36 | -11.49 | 18.26 | 3.04 | -51.89 | 21.17 |
