# Supplementary material for: Insufficient Stability of Clavulanic Acid in Widely Used Child-Appropriate Formulations
Source: Antibiotics (Basel). 2021 Feb 23;10(2):225. doi: 10.3390/antibiotics10020225 (PMC7927114; doi:10.3390/antibiotics10020225)
Supplement: Supplementary file 1 [file antibiotics-10-00225-s001.zip › Amox Co-amox stability_Mack_Table S5.docx]

**Table 5.** Degradation of Amoxicillin in non-co-formulated suspensions at 28°C and 8°C. Mean, median, standard deviation (sd), standard error (se), and 95% confidence interval (“lower” and “upper”) are reported for each of the two suspensions (Mepha and Sandoz).

| **days** | **temp** | **type** | **N** | **mean** | **median** | **sd** | **se** | **lower** | **upper** |
| --- | --- | --- | --- | --- | --- | --- | --- | --- | --- |
| 0 | 28°C | Mepha | 9 | 3.92 | 0.00 | 7.13 | 2.38 | -10.35 | 18.19 |
| 0 | 28°C | Sandoz | 9 | -1.66 | 0.00 | 5.46 | 1.82 | -12.59 | 9.26 |
| 0 | 28°C | both | 18 | 1.13 | 0.00 | 6.80 | 1.60 | -12.47 | 14.73 |
| 1 | 28°C | Mepha | 9 | 3.26 | 2.62 | 6.16 | 2.05 | -9.07 | 15.58 |
| 1 | 28°C | Sandoz | 8 | -11.88 | -14.17 | 9.41 | 3.33 | -30.69 | 6.94 |
| 1 | 28°C | both | 17 | -3.86 | -0.70 | 10.88 | 2.64 | -25.62 | 17.89 |
| 4 | 28°C | Mepha | 9 | 3.88 | 5.51 | 5.67 | 1.89 | -7.47 | 15.23 |
| 4 | 28°C | Sandoz | 9 | -0.19 | -1.75 | 8.69 | 2.90 | -17.56 | 17.19 |
| 4 | 28°C | both | 18 | 1.85 | 3.43 | 7.42 | 1.75 | -12.99 | 16.69 |
| 7 | 28°C | Mepha | 9 | -6.50 | -2.91 | 7.64 | 2.55 | -21.79 | 8.78 |
| 7 | 28°C | Sandoz | 9 | -11.54 | -12.59 | 6.12 | 2.04 | -23.79 | 0.70 |
| 7 | 28°C | both | 18 | -9.02 | -10.43 | 7.20 | 1.70 | -23.42 | 5.38 |
| **days** | **temp** | **type** | **N** | **mean** | **median** | **sd** | **se** | **lower** | **upper** |
| 0 | 8°C | Mepha | 9 | -0.43 | 0.00 | 6.09 | 2.03 | -12.62 | 11.75 |
| 0 | 8°C | Sandoz | 9 | -1.44 | 0.00 | 6.80 | 2.27 | -15.04 | 12.17 |
| 0 | 8°C | both | 18 | -0.94 | 0.00 | 6.29 | 1.48 | -13.51 | 11.64 |
| 1 | 8°C | Mepha | 9 | -4.63 | -3.37 | 6.42 | 2.14 | -17.47 | 8.21 |
| 1 | 8°C | Sandoz | 9 | 5.39 | 6.53 | 6.20 | 2.07 | -7.01 | 17.79 |
| 1 | 8°C | both | 18 | 0.38 | -0.07 | 8.00 | 1.89 | -15.63 | 16.39 |
| 4 | 8°C | Mepha | 9 | -10.24 | -8.65 | 6.71 | 2.24 | -23.65 | 3.18 |
| 4 | 8°C | Sandoz | 9 | 1.31 | -1.78 | 14.57 | 4.86 | -27.84 | 30.46 |
| 4 | 8°C | both | 18 | -4.46 | -6.99 | 12.51 | 2.95 | -29.48 | 20.55 |
| 7 | 8°C | Mepha | 9 | -0.44 | 0.54 | 12.73 | 4.24 | -25.90 | 25.02 |
| 7 | 8°C | Sandoz | 9 | 1.05 | 1.90 | 8.60 | 2.87 | -16.15 | 18.24 |
| 7 | 8°C | both | 18 | 0.30 | 1.48 | 10.56 | 2.49 | -20.83 | 21.43 |
