# Supplementary material for: Insufficient Stability of Clavulanic Acid in Widely Used Child-Appropriate Formulations
Source: Antibiotics (Basel). 2021 Feb 23;10(2):225. doi: 10.3390/antibiotics10020225 (PMC7927114; doi:10.3390/antibiotics10020225)
Supplement: Supplementary file 1 [file antibiotics-10-00225-s001.zip › Amox Co-amox stability_Mack_Table S6.docx]

**Table 6.** Degradation of Amoxicillin in non-co-formulated dispersible tablets at 28°C and 23°C. Mean, median, standard deviation (sd), standard error (se), and 95% confidence interval (“lower” and “upper”) are reported for the Sandoz 1000 mg tablet.

| **hours** | **temp** | **type** | **N** | **mean** | **median** | **sd** | **se** | **lower** | **upper** |
| --- | --- | --- | --- | --- | --- | --- | --- | --- | --- |
| 0.25 | 28°C | Sandoz | 9 | -2.68 | 0.00 | 7.87 | 2.62 | -18.41 | 13.05 |
| 1 | 28°C | Sandoz | 9 | 6.75 | 0.04 | 15.12 | 5.04 | -23.49 | 37.00 |
| 4 | 28°C | Sandoz | 9 | -1.00 | -0.21 | 8.94 | 2.98 | -18.88 | 16.89 |
| 8 | 28°C | Sandoz | 9 | 1.53 | 1.69 | 4.65 | 1.55 | -7.77 | 10.84 |
| 12 | 28°C | Sandoz | 9 | -3.03 | -3.27 | 4.52 | 1.51 | -12.07 | 6.00 |
| 24 | 28°C | Sandoz | 9 | 6.67 | 6.81 | 3.41 | 1.14 | -0.16 | 13.50 |
| **hours** | **temp** | **type** | **N** | **mean** | **median** | **sd** | **se** | **lower** | **upper** |
| 0.25 | 23°C | Sandoz | 9 | -1.78 | 0.00 | 9.82 | 3.27 | -21.43 | 17.87 |
| 1 | 23°C | Sandoz | 9 | -0.88 | 0.23 | 8.47 | 2.82 | -17.82 | 16.06 |
| 4 | 23°C | Sandoz | 9 | -11.49 | -11.23 | 5.22 | 1.74 | -21.93 | -1.04 |
| 8 | 23°C | Sandoz | 9 | -2.58 | -3.21 | 7.14 | 2.38 | -16.86 | 11.70 |
| 12 | 23°C | Sandoz | 9 | -3.01 | -3.80 | 5.56 | 1.85 | -14.14 | 8.11 |
| 24 | 23°C | Sandoz | 9 | -6.92 | -10.01 | 6.62 | 2.21 | -20.15 | 6.31 |
