# Supplementary material for: Insufficient Stability of Clavulanic Acid in Widely Used Child-Appropriate Formulations
Source: Antibiotics (Basel). 2021 Feb 23;10(2):225. doi: 10.3390/antibiotics10020225 (PMC7927114; doi:10.3390/antibiotics10020225)
Supplement: Supplementary file 1 [file antibiotics-10-00225-s001.zip › Amox Co-amox stability_Mack_Table S7.docx]

**Table 7.** Percentage (%) of households in represented African and Asian countries having access to electricity or refrigeration, assessed during DHS or MIS surveys from 2014-2018.

| **Country** | **Year/Survey** | **Electricity**  **Total (%)** | **Refrigerator**  **Total (%)** | **Average Dry Bulb Temperature (°C)** |
| --- | --- | --- | --- | --- |
| Afghanistan | 2015 DHS | 71.5 | 19.2 | 14.5 |
| Angola | 2015-16 DHS | 41.6 | 35.5 | 23.6 |
| Bangladesh | 2014 DHS | 62.4 | 20.2 |  |
| Benin | 2017-18 DHS | 35.6 | 4.6 | 27.3 |
| Burkina Faso | 2017-18 MIS | 16.8 | 5.8 |  |
| Burundi | 2016-17 DHS | 8.7 | 1.4 |  |
| Cambodia | 2014 DHS | 56.1 | 8 |  |
| Chad | 2014-15 DHS | 7.7 | 2 |  |
| Ethiopia | 2016 DHS | 25.6 | 5.3 |  |
| Ghana | 2016 MIS | 79.3 | 34.7 |  |
| India | 2015-16 DHS | 88.2 | 29.6 | 25.3 |
| Kenya | 2015 MIS | 41.6 | 8 | 24.2 |
| Lesotho | 2014 DHS | 27.8 | 22 |  |
| Liberia | 2016 MIS | 19.8 | 7.7 |  |
| Madagascar | 2016 MIS | 22.9 | 3.5 |  |
| Malawi | 2017 MIS | 12.6 | 5.1 | 21.4 |
| Maldives | 2016-17 DHS | 99.8 | 97.8 |  |
| Mali | 2015 MIS | 37.6 | 9.5 | 28.3 |
| Mozambique | 2018 MIS | 27.9 | 17.1 |  |
| Myanmar | 2015-16 DHS | 55.6 | 16 |  |
| Nepal | 2016 DHS | 90.5 | 15.5 |  |
| Nigeria | 2015 MIS | 52.2 | 23 | 26.8 |
| Pakistan | 2017-18 DHS | 92.7 | 55.4 | 22.7 |
| Philippines | 2017 DHS | 92.7 | 42.7 | 27.3 |
| Rwanda | 2017 MIS | 33.2 | 1.7 |  |
| Senegal | 2017 DHS | 63.3 | 28.3 |  |
| Sierra Leone | 2016 MIS | 20.3 | 11.9 | 26.2 |
| South Africa | 2016 DHS | 90 | 74.7 | 16.5 |
| Tanzania | 2017 MIS | 25.8 | 7.9 |  |
| Timor-Leste | 2016 DHS | 73.3 | 19.6 |  |
| Togo | 2017 MIS | 51.6 | 7.7 | 27.1 |
| Uganda | 2016 DHS | 28.6 | 5.6 |  |
| Zimbabwe | 2015 DHS | 33.7 | 24.8 | 18.8 |

Note: Average dry bulb temperature: From UNDATA, based on World Meteorological Organization data, http://data.un.org/Data.aspx?q=temperature&d=CLINO&f=ElementCode%3a87#CLINO (accessed 04 March 2019) Abbreviations: DHS, Demographic and Health Survey; MIS, Malaria Indicator Survey.
